# A Clinical and Radiographic Paradox in a Case of Barium Aspiration

**DOI:** 10.7759/cureus.28370

**Published:** 2022-08-25

**Authors:** Sankalp Yadav

**Affiliations:** 1 Emergency Medicine, Shri Madan Lal Khurana Chest Clinic, New Delhi, IND

**Keywords:** barium swallow, covid-19, oropharyngeal dysphagia, gastroesophageal reflux disease (gerd), barium sulphate aspiration

## Abstract

Barium studies are essential to the diagnostic work-up, especially in dysphagia, heartburn (dyspepsia), or gastroesophageal reflux (GERD). The barium swallow test, or esophagram, is essential in investigating patients with abnormalities within the esophagus. The barium sulfate used is inert to the gastrointestinal tract and causes no harm. However, problems begin when the contrast agent enters the trachea and then into the lungs. The author herein presents a case of barium aspiration in a 60 years old Indian male being investigated for the cause of dysphagia. In this case, the paradox was the radiograph's contradictory features and the patient's clinical features. The large aspiration on the radiograph alerted the clinicians. However, the patient was relatively asymptomatic, and thus a clinical and radiographic paradox should always be kept in the minds of the treating physicians, especially post barium aspiration.

## Introduction

Cases of aspiration pneumonia are common [1]. Most of these cases occur in healthy asymptomatic individuals, are self-limiting, and require no treatment [1]. The issue becomes necessary when the aspiration is life-threatening [2]. The barium swallow test is a standard test routinely done to determine the common issues related to the gastrointestinal (GI) tract [2]. Barium sulfate is a contrast that is inert and causes no harm to the GI tract [3]. However, this simple test could threaten life if the barium is accidentally aspirated [4]. There are several cases of barium aspiration available in the literature. In most of these cases, the patients were asymptomatic, but reports of life-threatening effects of barium aspiration are also well documented [4].

Herein, a case of an elderly Indian male who had barium aspiration during a diagnostic work-up for dysphagia is presented. The extensive aspiration was alarming on the radiograph, but the patient was relatively asymptomatic except for a mild cough with white expectoration; therefore, this case emphasizes the clinical and radiographic paradox in such scenarios that should always be in the back of the minds of the treating clinicians.

## Case presentation

A 60-year-old male came to the outpatient department (OPD) as a referred case from the department of ENT with complaints of cough with white expectoration for one hour. He was undergoing a diagnostic work-up for the dysphagia during which he accidentally aspirated barium sulfate during the barium swallow studies. Dysphagia was suspected to be due to esophageal motility (movement) issues or age. The procedure was immediately terminated.

**Figure 1:**
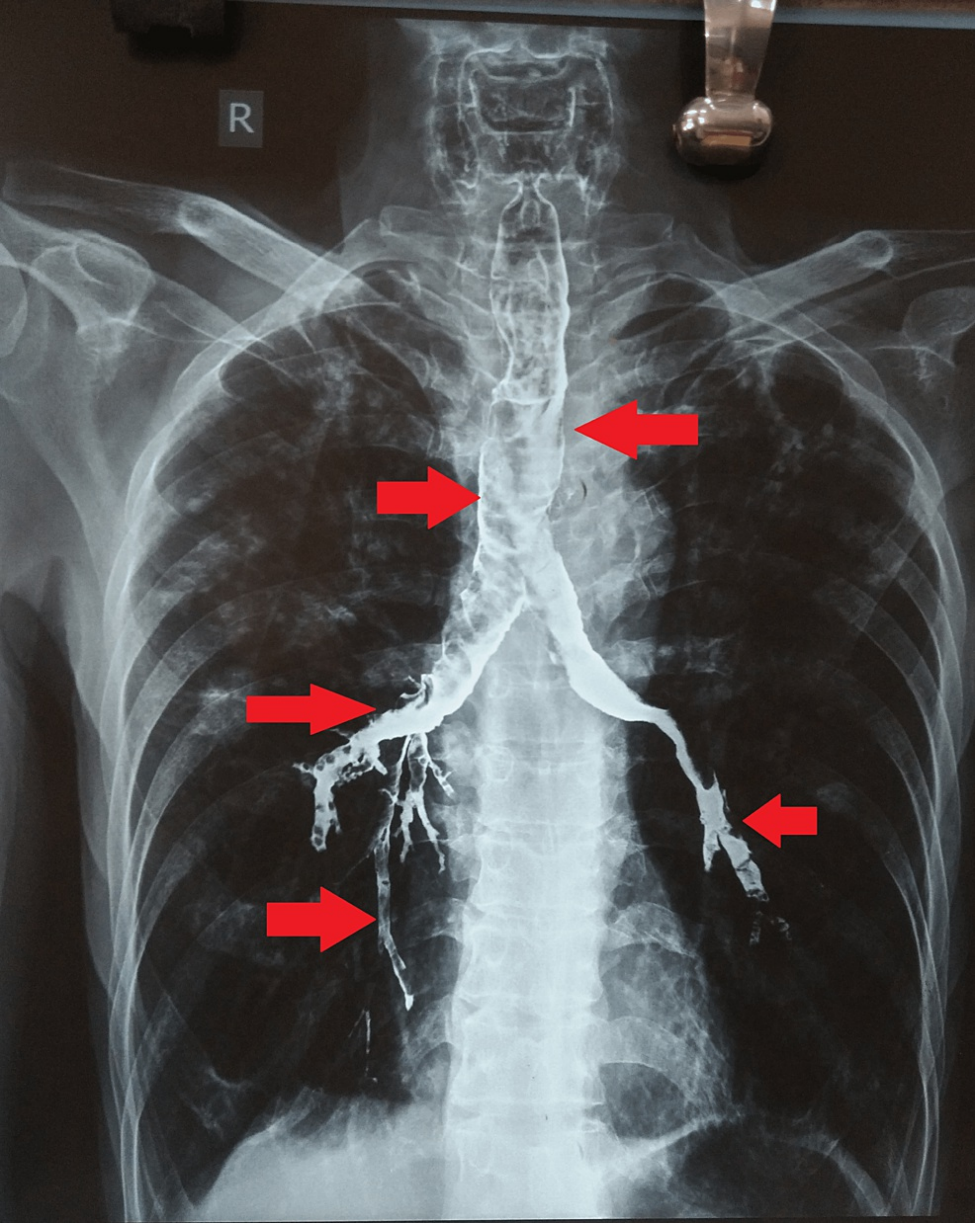
Post-barium swallow chest radiograph (P-A view)

The cough began after the aspiration of the barium sulfate; however, it subsided after one and half hours. However, he continued to produce a white expectoration with an occasional cough. His vitals were pulse- 80/minute, blood pressure- 130/80 mmHg, respiratory rate- 21/minute, SpO2- 96% on room air, and temperature-98.4 degrees Fahrenheit. He was conscious and fully oriented to time, place, and person on examination. The Glasgow coma scale score was 15/15. Systemic examination was unremarkable. Detailed laboratory work-up was not suggestive of any abnormalities. The post-barium swallow chest radiograph (P-A view) (Figure 1) was suggestive of aspiration of radio-opaque material consistent with barium in the trachea, both main bronchi, and the lungs. The aspiration was more on the right middle and lower lobes than the left lower lobes. He was admitted for monitoring of vitals with a direction to intubate in case of respiratory distress. Chest physiotherapy with postural drainage techniques was performed, which helped him increase white expectoration production. Bronchoalveolar lavage was not required.

Further, he was stable throughout the admission, and there were no dyspnea or respiratory distress episodes. His vitals were normal, with SpO2 98% on room air. Considering the pandemic of COVID-19 with oversaturated health facilities and limited bed availabilities, he was discharged the next day with advice to follow up in the OPD. On two follow-ups, he continued to do well with dysphagia attributed to his age with no significant complaints and ultimately was lost-to-follow-up.

## Discussion

The use of radiographic contrast in studies to determine the causes of dysphagia is widespread [5]. Barium is commonly used in dysphagia studies as high-osmotic water-soluble agents risk pulmonary edema [5]. However, problems arise when the patient accidentally aspirates the contrast agent. Aspiration of radiographic contrast like barium sulfate during investigations is possible and could be life-threatening [4,5]. Barium aspiration is rare and usually benign in most cases [6,7]. But severe complications like anaphylaxis, airway obstruction, aspiration pneumonia, clinical decompensation, and/or respiratory failure are documented in the literature [8]. Massive aspirations lead to a mechanical obstruction, thereby increasing the alveolar dead space resulting in an altered ventilation/perfusion (V/Q) ratio with secondary respiratory failure and could result in fatal outcomes [9].

The possibilities of contrast aspiration are higher in certain conditions like extremes of age, low level of consciousness, the anatomical and functional integrity of the oropharyngeal and esophageal segments, broncho-oesophageal fistula, alcoholism, neuromuscular dysfunction, disordered swallowing, head and neck cancer, and psychological illness usually associated with functional gastrointestinal disorders [9]. Moreover, the mortality rate after massive barium aspiration ranges from 30% and could exceed 50% in patients with complications like initial shock or apnea, secondary aspiration pneumonia, or adult respiratory distress syndrome [10].

A case similar to this was published by Pata et al., where the patient was mainly asymptomatic and did not require intubation. However, the present case differs from their case in age and bilateral aspiration of the barium sulfate [2]. However, this case shares similarities with their case in a clinical and radiographic paradox where the chest radiograph was suggestive of aspiration, but the patient was clinically stable [2].

Another case similar to the present case was published by Kumar et al.. However, this case differs from their index case in clinical presentation, location of the aspirated contrast in the lungs, and management where bronchoalveolar lavage was not required [9]. Compared to the case of Kumar et al., the present case was asymptomatic, with no complications like respiratory distress [9].

The management of barium aspiration is mainly supportive. It involves early identification of predisposing factors, pretreatment with antireflux medications, such as domperidone, and retro esophageal suction catheter during barium swallow study [9]. Treating physicians must evaluate the volume and dose of barium sulfate and look for alternative options in those with predisposing risk factors [8]. In the absence of a definite supportive therapy, including supplemental oxygen, IV fluids, and antibiotics for superimposed infections [8]. Further, in barium use procedures, lateral projection fluoroscopy of the pharyngeal phase of swallowing should be considered [5]. Reports of early bronchoalveolar lavage, especially in cases with mechanical obstruction resulting in an altered V/Q ratio with an impending secondary respiratory failure, are also available in the literature [9]. However, the use of bronchoalveolar lavage is not recommended in studies by Tamm and Kortsik due to the potential danger of disseminating the barium into the bronchioalveolar system [9]. There is a paucity of data from long-term follow-ups in cases of barium sulfate aspiration. Thus large-scale studies aimed at studying the delayed impact of a contrast agent aspiration are essential. Furthermore, in the absence of no uniform management guidelines, paucity of data treatment should be based on clinical judgment and common sense [9].

## Conclusions

The author reports a case of rare, accidental aspiration of contrast material, i.e., barium sulfate, in an elderly Indian male. The radiographs post barium swallow procedure suggests a bilateral aspiration that could have resulted in intubation. However, the patient had a mild cough with white expectoration and remained asymptomatic on admission and monitoring. The present case is thus a clinical and radiographic paradox where the chest radiograph was dramatic with the extent of aspiration, but the patient was clinically stable with minimal symptoms. This case also emphasizes the need for judicious use of resources, especially during an ongoing pandemic of COVID-19, as unnecessary intubation based on radiographical findings could have led to complications.
